# An Epidemiological Framework for Modelling Fungicide Dynamics and Control

**DOI:** 10.1371/journal.pone.0040941

**Published:** 2012-08-10

**Authors:** Matthew D. Castle, Christopher A. Gilligan

**Affiliations:** Department of Plant Sciences, University of Cambridge, Cambridge, United Kingdom; Institute for Animal Health, United Kingdom

## Abstract

Defining appropriate policies for controlling the spread of fungal disease in agricultural landscapes requires appropriate theoretical models. Most existing models for the fungicidal control of plant diseases do not explicitly include the dynamics of the fungicide itself, nor do they consider the impact of infection occurring during the host growth phase. We introduce a modelling framework for fungicide application that allows us to consider how “explicit” modelling of fungicide dynamics affects the invasion and persistence of plant pathogens. Specifically, we show that “explicit” models exhibit bistability zones for values of the basic reproductive number (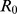) less than one within which the invasion and persistence threshold depends on the initial infection levels. This is in contrast to classical models where invasion and persistence thresholds are solely dependent on 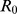. In addition if initial infection occurs during the growth phase then an additional “invasion zone” can exist for even smaller values of 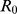. Within this region the system will experience an epidemic that is not able to persist. We further show that ideal fungicides with high levels of effectiveness, low rates of application and low rates of decay lead to the existence of these bistability zones. The results are robust to the inclusion of demographic stochasticity.

## Introduction

Fungicide use is an essential aspect of disease management in modern agriculture [1]. There are, however, increasing restrictions upon the use of fungicides (and other forms of chemical control) [2], [3] and the trend is for reduced usage, to avoid undue release to the environment or to minimise the risk of fungicide resistance. Reduction in the availability and use of fungicides imposes greater demands upon efficient use of the limited resources available. One way to approach this is by the use of mathematical models to investigate the effects of a given fungicide on the host crop and the pathogen to aid the design of appropriate disease management strategies [4]–[6]. The benefits of modelling diseases are well acknowledged: it allows theoretical optimal control strategies to be developed that minimise economic costs and maximise crop returns [7]–[10], which can then be tested experimentally [11]. Furthermore the continued widespread use of fungicides is threatened by the emergence of resistant pathogen strains, often as a direct consequence of the application strategy itself [1], [2], [12] and in order to implement effective resistance management strategies it is often necessary to model those strategies first [9], [13]–[16].

Most models that include fungicides often account for fungicide dynamics by simply modifying the parameters of the underlying epidemiological models (i.e. reducing the infection rates and/or increasing the host recovery rates) [9], [14], [17]. Much work has been done to investigate the consequences of using different underlying models for both the host population and pathogen dynamics (see [6] for a review) but relatively little work has been carried out to investigate the dynamics of the fungicides themselves and how the timing of initial infection relative to host population growth affects invasion and persistence in a chemically controlled system. Generally, previous work has implicitly made one or more of the following assumptions; that there is complete coverage by the fungicide for either the entire host population or a fixed subset of the host population [10], [14], [16], [18], [19]; that a generic, multi-purpose, fungicide has been applied to the hosts (i.e. they have not readily separated out the effects of different fungicide types such as protectants, curatives or eradicants) [9], [20], [21]; or that the fungicides are permanent (i.e. that the chemicals do not decay or that their effects do not change over time) [18].

Here we propose a parsimonious model framework that is designed to integrate epidemiological and fungicide dynamics. Specifically we consider purely protectant fungicides, and we distinguish the effects of the rate of application, the decay in activity and the partial effectiveness of the fungicide on the epidemic dynamics. We first use the framework to construct a deterministic compartmental model that incorporates host growth and explicitly allows for the dynamic application of a purely protectant fungicide through the use of multiple susceptible host classes. We compare this model with a conventional model that implicitly incorporates the protectant fungicide dynamics through modification of the underlying infection rates for a single susceptible host class. For brevity, we refer to these two models as the explicit and implicit models respectively. We then extend the explicit model to include demographic stochasticity.

Given that the application of fungicides is usually designed to prevent invasion of a pathogen or to eliminate the pathogen, we use the two models to ask the following questions:

- does the explicit inclusion of fungicide dynamics affect the criteria for invasion and persistence?
- does the timing of initial infection affect the criteria for invasion and persistence in a chemically controlled system?
- how are the differences manifested?
- does failure to account for fungicide dynamics lead to erroneous predictions of control effectiveness in epidemiological models?
- are there critical regions of parameter space concerned with coverage, decay and effectiveness of fungicides for which these differences are most exaggerated?
- are the differences maintained when allowance is made for stochastic variability in the transmission of infection?

## Methods

### Deterministic Model Derivation

Consider a pathogen spreading through a population of hosts such as a single field of a crop, in which the epidemiological host unit is an amount of plant tissue i.e. a leaf, stem, or root [4] and there is a simple density dependent growth of the host up to a carrying capacity. In the absence of any form of chemical control the population is divided into two classes, susceptible (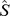) and infected (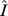). We assume that infected host tissue consumes resources but does not contribute to new host growth [22], that infection is adequately described as a mass action process (this assumption holds well for pathogens within areas smaller than their average dispersal distance [23]) and that hosts cannot be re-infected after they cease to be infectious, and are removed. We investigate the effects of applying a purely protectant fungicide to the system. Here we assume that a protectant fungicide affects susceptible hosts, reducing their capacity to become infected.

**Figure 1:**
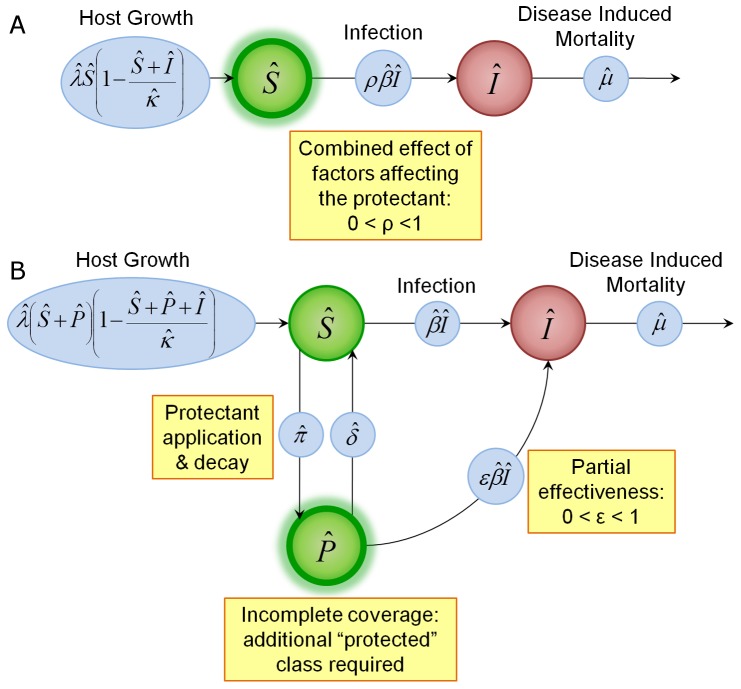
Model transtion diagrams. (A) The conventional model (the ‘implicit’ model) incorporates fungicide dynamics implicitly and therefore assumes uniform, permanent coverage by the protectant. (B) The model with explicit fungicide dynamics (the ‘explicit’ Model) allows for partial coverage and decay in activity of the protectant as well as different infection rates depending on whether the hosts have a protectant fungicide applied or not. The variables and parameters are explained in Table 2.

We first consider a conventional model that only includes implicit fungicide dynamics, we assume that all susceptible hosts are protected. All factors affecting the effectiveness of the protectant are subsumed into a single parameter (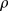) which represents the average decrease in the infection rate for the host population. The dynamics of this model are represented in Figure 1 and by the equations:
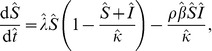
(1)

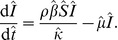
(2)


Here, density dependent host growth is governed by the rate 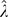 and a carrying capacity 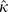. 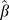 represents the infection rate in the absence of chemical control. The parameter 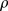 (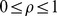) is a measure of fungicide effectiveness. It reduces the infection rate and represents the combined effects of the partial coverage, decay in activity and incomplete effectiveness of the protectant fungicide (possible expressions for 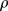 that reflect different combinations of these effects are summarised in Table 1). Infected hosts are removed at a per capita rate (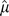).

**Table 1:** Expressions for 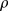. Potential expressions for 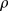, the reduction in infection rate in the implicit model, and their interpretations in terms of assumptions made about the behaviour of the protectant.

Functional Form For 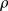	Interpretation
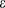	1. Complete coverage of hosts. 2. No decay of protectant. 3. Protectant is partially effective
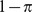	1. Fixed proportion of hosts covered 2. No decay of protectant 3. Protectant is completely effective
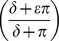	1. Fixed equilibrium proportion of hosts covered 2. Protectant is allowed to decay 3. Protectant is partially effective

We choose an expression for 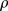 that takes account of the uninfected population being an aggregation of completely unprotected and partially protected hosts. Given a protectant application rate (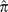) and a protectant decay rate (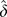) the long term proportions for unprotected and protected hosts are 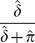 and 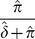 respectively. In the absence of explicit treatment of fungicide dynamics, we assume these proportions are maintained throughout the infection process, and that the protected hosts are infected at a reduced per capita rate (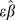, 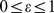), where 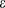 is a measure of the effectiveness the protectant fungicide (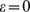 and 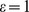 correspond to completely effective and ineffective protectant fungicides respectively). The total rate of infection is therefore
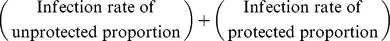


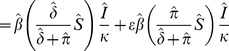


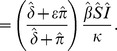



As such we take 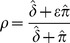, which yields our conventional, ‘implicit’ model:
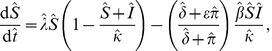
(3)

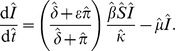
(4)


In order to incorporate fungicide dynamics explicitly we propose an alternative model with an additional protected host class (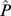). Here, the per capita protectant application rate (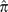) and the per capita protectant decay rate (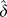) allow individuals to move between the susceptible (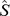) and protected (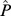) states. The protected state experiences reduced infection rates (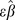) whilst the unprotected susceptible state experiences normal infection rates (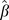). New host units are assumed to be unprotected which is consistent with a non-systemic protectant fungicide. The dynamics of this model are represented in Figure 1 and by the equations:
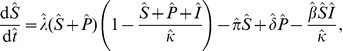
(5)

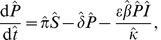
(6)

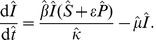
(7)


#### Non-dimensionalisation

We introduce the dimensionless variables
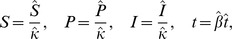
and parameters
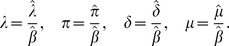
The conventional model equations (1) and (2) with implicit fungicide dynamics become
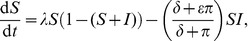
(8)

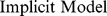


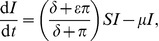
(9)and the alternative model equations (5), (6) and (7) with explicit fungicide dynamics become
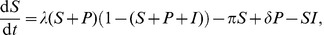
(10)

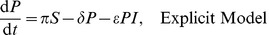
(11)

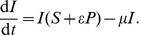
(12)


Henceforth we refer to the conventional model as the implicit model and the alternative model as the explicit model. The parameters, their definitions, and typical values used for the numerical simulations are summarised in Table 2. The default values used hereafter are chosen primarily to highlight the characteristic properties of the model system, however the parameter ratios are consistent with those used in disease management practices for both barley and wheat crops [24]–[27].

**Table 2:** Parameter Summary. Summary of dimensionless state variables, initial conditions, equilibria values and system parameters for both the implicit and explicit models.

Variable	Definition	Description	Default Value
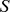	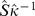	Density of susceptible hosts	-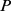	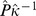	Density of protected hosts	-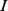	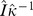	Density of infected hosts	-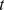	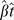	Time	-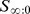	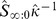	Disease free equilibrium density of susceptible hosts	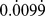
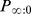	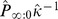	Disease free equilibrium density of protected hosts	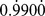
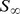	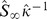	Endemic equilibrium density of susceptible hosts	varies
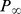	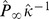	Endemic equilibrium density of protected hosts	varies
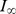	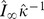	Endemic Equilibrium density of infected hosts	varies
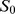	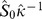	Initial density of susceptible hosts at infection	varies
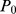	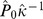	Initial density of protected hosts at infection	varies
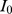	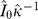	Initial density of infected hosts	varies
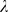	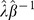	Growth rate of hosts	0.5
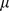	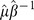	Removal rate	varies
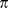	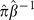	Protectant application rate	0.1
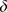	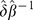	Protectant decay rate	0.001
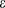	-	Protectant effectiveness	0.1
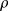	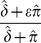	Reduction in infection rate (implicit model)	0.109
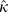	-	Disease free carrying capacity	2000
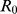	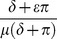	Basic reproductive number	varies

### Stochastic Model

In order to demonstrate that existence of the bistability zone is robust to demographic stochasticity and that it is not just a property of the deterministic nature of the explicit model, a continuous-time Markov process version of the explicit model is constructed.

Again 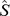, 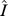 and 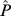 represent the actual numbers of hosts in each of the susceptible, infected and protected classes. Given a carrying capacity, 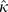, the infinitesimal transition probabilities that describe the Markov process are given in Table 3. For computational efficiency a continuous-time Gillespie algorithm [28] is used to generate the sequence of transition event times for each simulation and thus obtain the trajectories for each class.

**Table 3:** Transition Probabilities. Infinitesimal transition probabilities for the stochastic Markov process version of the explicit model.

Transition Event	Infinitesimal Transition Probability
Net Birth	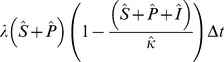
Susceptible Infection	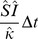
Protected Infection	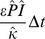
Protectant Application	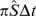
Protectant Decay	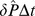
Removal	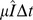
No Event Happens	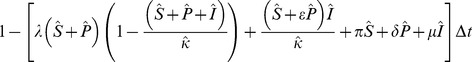

## Results

### Equilibrium Analyses

Both the implicit model and the explicit model have the same basic reproductive number for the pathogen:
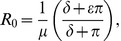
(13)with the disease free equilibria 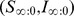 and 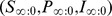 for the models given by
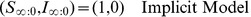
(14)

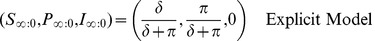
(15)


It is trivial to show that both disease free equilibria are stable for 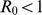 and unstable for 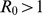. The implicit model only admits a single endemic equilibrium 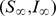, given by:
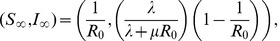
(16)whereas the explicit model admits multiple endemic equilibria, given by:
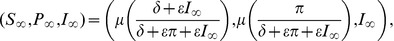
(17)where 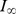 is any root to the cubic equation:
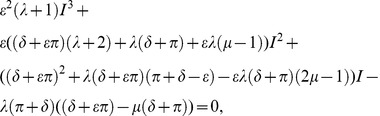
(18)that satisfies 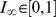.

**Figure 2:**
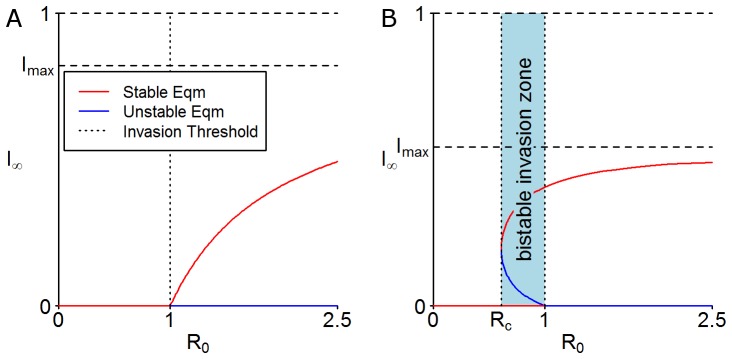
Model bifurcation diagrams. Bifurcation diagrams for the implicit and explicit models showing the effect of explicitly including fungicide dynamics on the endemic equilibria of the system. (A) The implicit model only admits bifurcation diagrams with a single invasion threshold at 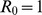. (B) The explicit model can additionally admit a bistable invasion zone. Within this zone invasion depends upon the initial level of infection (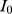). The default parameters used for these plots are given in Table 2.

It can be shown that the endemic equilibrium for the implicit model is both biologically meaningful and stable only for 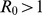. This model gives rise to a classical epidemiological bifurcation diagram (a graph of 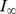 as a function of 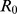 showing a transcritical bifurcation at 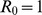) given by Figure 2 and we see a single invasion threshold at 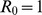 as expected.

Allowance for fungicide dynamics in the explicit model yields zero, one, or two biologically realistic endemic equilibria depending on the values of the model parameters 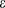, 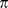, 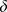 and 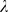. In the region where 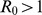 there is only ever one single, stable, endemic equilibrium. However, in the region where 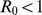 the other parameters affect the number of possible equilibria. Let
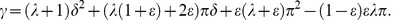
(19)


If 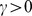 then there is no biologically meaningful (stable or unstable) endemic equilibrium for 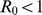. This again gives rise to a classical epidemiological bifurcation diagram. If, however, 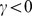, then a second threshold 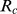 exists and it is possible for the system to have two endemic equilibria for 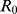 between 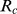 and 1 (Figure 2). The lower branch of the endemic equilibrium curve is always unstable and the upper branch stable [29]. It follows that failure to account explicitly for fungicide dynamics can lead to an erroneous understanding of the nature of the system. In particular, explicitly accounting for fungicide dynamics means that for values of 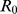 less than 1, it is still possible for infection to persist within the system.

**Figure 3:**
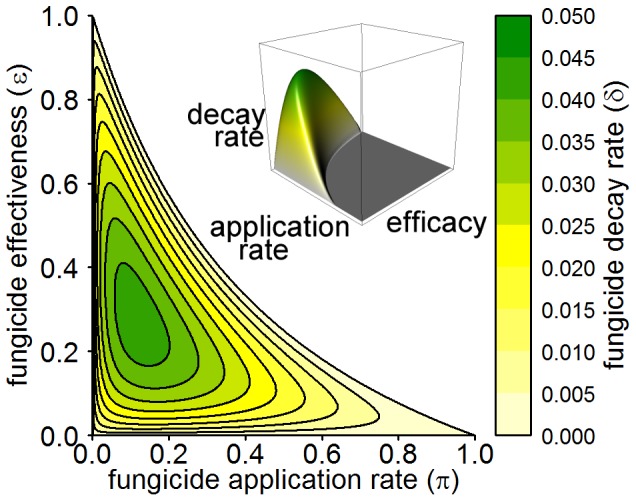
Critical fungicide properties required for bifurcation. A contour plot for critical fungicide properties (efficacy, application rate and decay rate) that defines the region where there is a bistable invasion zone. For values of 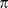, 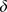 and 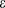 that lie within this region, the system exhibits a backwards bifurcation and a pathogen is able to invade and persist for values of 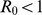. For parameter values outside this region the system exhibits a traditional bifurcation diagram and a pathogen is only able to invade for 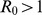. Inset is a 3D perspective plot of the same surface. For these plots the growth rate of the host, 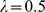.

The threshold 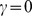 can be interpreted as a surface in the fungicide parameter (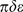) space with 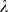 fixed (see Figure 3). Here we note that if the fungicide parameters lie in the region below this surface then the model exhibits bistability. The surface encloses the region of fungicide parameter space near to the origin, i.e. low values of the scaled parameters (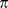, 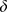 and 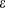). It is a key result to note that these values correspond to highly effective fungicides (low 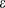), with long lifespans (low 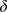) that are applied infrequently (low 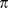) and so it follows that a move towards using fungicides with these properties can have extremely undesirable consequences in terms of effective control.

### Invasion and Persistence Criteria

We now consider how invasion and persistence criteria depend on the choice of model and on the host growth state of the system at the time of initial infection. In particular we determine how these criteria depend on both the basic reproductive ratio 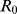 and the initial levels of infection (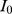). Invasion is related to the immediate behaviour of the system, and a pathogen is considered to have invaded if there is an immediate increase in the initial levels of infection. Persistence is related to the long term behaviour of the system, and a pathogen is considered to persist if the infection levels reach stable endemic equilibrium values.

#### Disease Free Host Growth

In the absence of infection the implicit and explicit models become
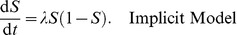
(20)

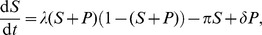


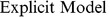
(21)

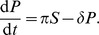



**Figure 4:**
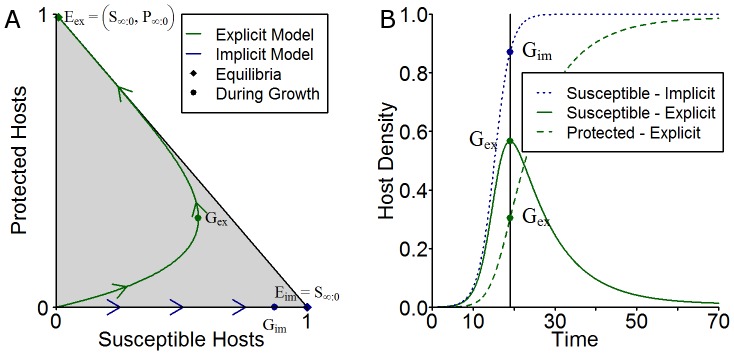
Disease-free growth dynamics. (A) A state space plot of the relative densities of susceptible (S) and protected (P) hosts during the disease free growth period for the explicit and implicit models. The points 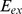 and 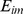 correspond to the disease-free equilibrium (DFEQ) densities for the explicit and implicit models respectively, and the points 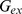 and 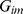 correspond to non-equilibrium densities (i.e. densities during the growth stage) of the disease-free states of the explicit and implicit models. (B) The equivalent plot of the densities of susceptible and protected hosts against time for the explicit model along with the equivalent growth curve of susceptible host density for the implicit model. The parameter values used here are given in Table 2.

Both models assume the same logistic growth of uninfected hosts up to a disease free equilibrium carrying capacity. However for the explicit model the relative levels of susceptible and protected hosts will vary during this growth phase (see Figure 4). Analytic solutions exist to both of these sets of equations.

#### Infection Choice

For each value of 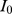 we must choose which uninfected hosts become infected. For the implicit model there is a single uninfected class and so the initial infected individuals must come from this class. If, prior to infection, the system has 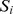 susceptible individuals, where 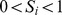 then immediately post infection the system must be in the state:
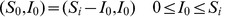
(22)


For the explicit model, with two uninfected classes, a choice is required. We assume that the proportion of 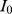 that comes from each uninfected class is proportional to their relative susceptibilities (i.e. more initial infections come from the susceptible class than the protected class) and their relative densities. i.e. If, prior to infection, the system is in state 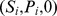 and immediately post infection the system is in state 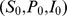 then
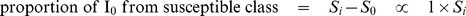


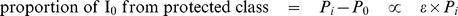



Consequently, for an initial level of infection, 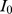, the initial post infection state 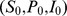 is given by:
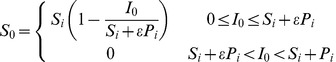
(23)

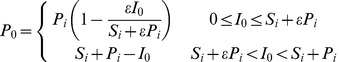
(24)


This gives us a method for moving from a pre-infection system state 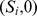 or 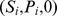 to an initial post-infection state 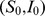 or 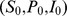 for the implicit and explicit models respectively.

#### Invasion and Persistence Thresholds

For a pathogen to invade we require that the level of infection increases immediately from the post-infection system state. For the implicit model this requires
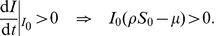
(25)


We use the relationship between pre-infection levels and post-infection levels to create a threshold in 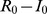 parameter space for each pre-infection susceptible host level 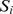. The threshold is given by:
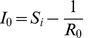
(26)


This reduces to the classic invasion threshold if 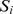 is taken to be the disease free equilibrium level 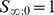. However for the explicit model we require:
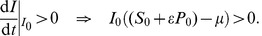
(27)


This gives the following threshold in 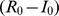 parameter space for each pre-infection host state 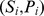:
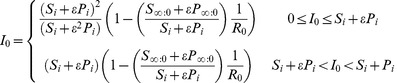
(28)


In the absence of analytical tractability persistence thresholds for the two models were determined numerically for a range of pre-infection states. The parameter values used for the numerical calculation are summarised in Table 2.

**Figure 5:**
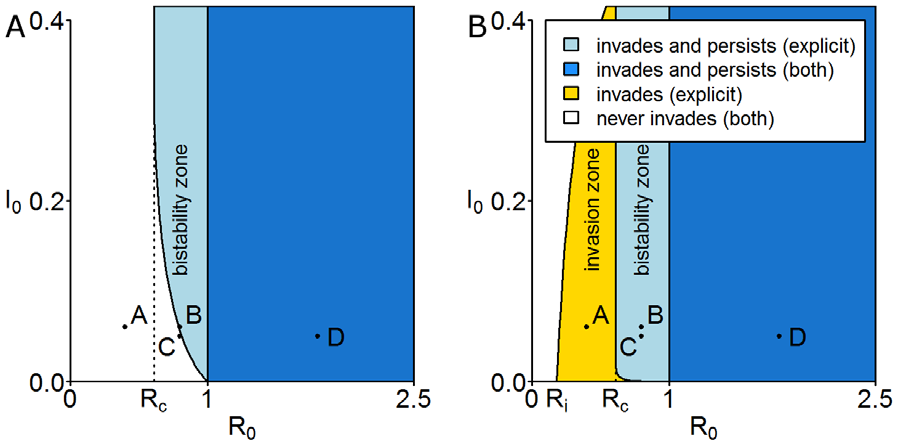
Invasion and persistence plots. Here we show the effects of 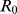 and initial level of infection at invasion (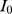) on invasion and persistence of the pathogen. (A) Pathogen invades system at disease free equilibrium (points 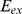 and 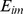 in Figure 4): for given 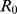 and 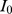 values the plot shows the long-term behaviour of both models in terms of the ability of a pathogen to invade and persist. For the implicit model 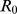 completely characterises the long-term behaviour of the system (with 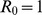 being the threshold). For the explicit model, the ability of a pathogen to invade and persist is also determined by initial level of infection, 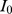, for a range of 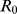 values (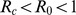). (B) Pathogen invades system during growth phase (points 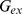 and 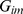 in Figure 4): For the implicit model 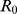 still completely characterises the long-term behaviour of the system. For the explicit model the bistability zone is extended and lower initial levels of infected hosts (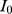) are able to invade and persist within the same range of 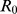 values (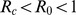). In addition, an extra invasion zone now exists for a range of values of 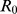 (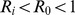) where here 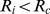. Within this zone the pathogen is able to invade but is not able to persist. Initial conditions for selected numerical simulations are shown. A:(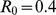, 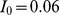) is within the invasion zone for the explicit model starting during the growth phase. B:(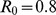, 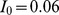) is just above the invasion and persistence threshold for the explicit model staring from the disease free equilibrium. C:(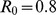, 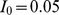) is just below the same threshold. D:(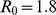, 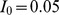) is significantly above the invasion and persistence thresholds for both models. The default parameter values are given in Table 2.

Using the results from the growth curves in section 0 and Figure 4 we illustrate how both the invasion and persistence thresholds depend upon the inclusion of fungicide dynamics for two distinct pre-infection states: the disease free equilibrium (points 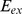 and 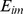 in Figure 4) and a pre-infection state during where the uninfected hosts are still growing (points 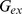 and 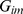 in Figure 4). The thresholds obtained are shown in Figure 5. We conclude that the explicit inclusion of fungicide dynamics leads to a zone of bistability for values of 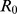 less than 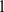, and that this zone is not simply an artefact of the equilibrium analysis but is robust to the system's transient dynamics. Furthermore we show that for pre-infection states where the host population is still growing the zone of bistability is extended and lower initial levels of infected hosts (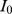) are able to invade and persist within the same range of 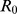 values. In addition an extra invasion zone now exists for a range of values of 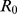 below 1. Within this invasion zone, a pathogen is able to invade but not persist (see Figure 5).

#### Deterministic Realisations

**Figure 6:**
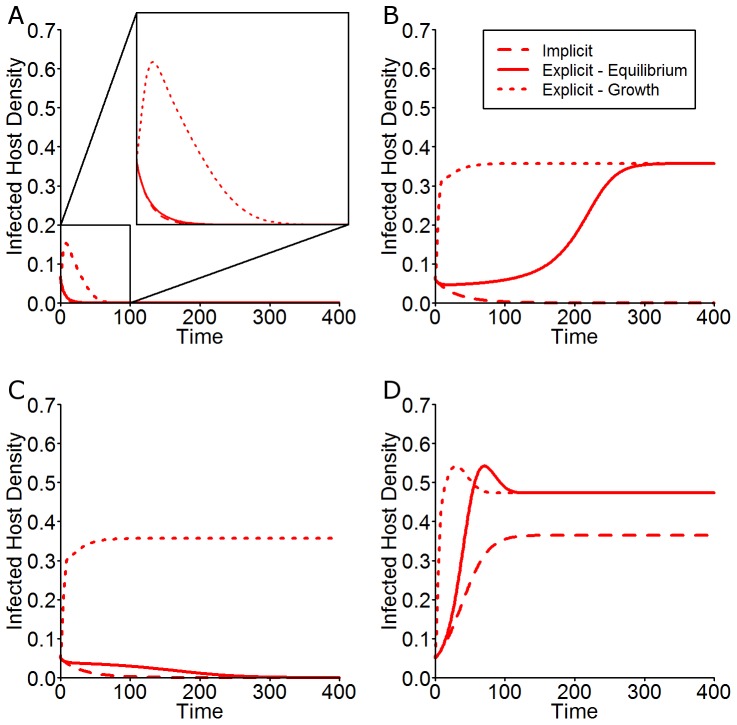
Deterministic realisations for the implicit and explicit models. These plots correspond to the initial conditions in the 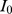-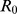 parameter space shown in Figure 5. (A) For a trajectory starting at point A (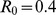, 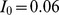) both models predict the long term extinction of the pathogen but the explicit model predicts invasion of the pathogen for a pre-infection state given by point 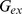 in Figure 4. (B) For a trajectory starting at point B (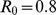, 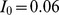) the implicit model predicts extinction, whereas the explicit model predicts persistence of the pathogen for both pre-infection states. (C) Both models predict the extinction of the pathogen for a system at disease-free equilibrium but the explicit model predicts the long-term persistence of the pathogen for a pre-infection state given by point 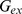 in Figure 4. (D) Both models predict the persistence of the pathogen sarting from point D (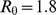, 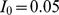), regardless of the pre-infection state. The default parameter values are given in Table 2.

Invasion trajectories for both models are shown in Figure 6. For initial conditions at point A (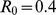, 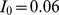), a value of 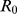 is chosen so that 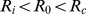 for the explicit model lies within the invasion zone for a pre-infection state with growing hosts. By considering the trajectories that result from this point it can clearly be seen that for the implicit model the choice of pre-infection state affects the pathogen's ability to invade. For initial conditions at points B (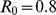, 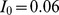) and C (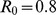, 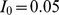), a value of 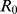 is chosen so that 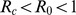 for the explicit model parameters. By considering the trajectories that result from these two points it can clearly be seen that for the implicit model the choice of initial infection level does not affect the ability of the pathogen to persist, whereas for the explicit model the initial infection level does affect the ability of the pathogen to persist. For initial conditions at point D (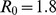, 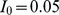) we can see that both models predict invasion for 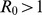, but that they do not agree on the final endemic level of infection, with the explicit model predicting higher levels.

#### Stochastic Realisations

**Figure 7:**
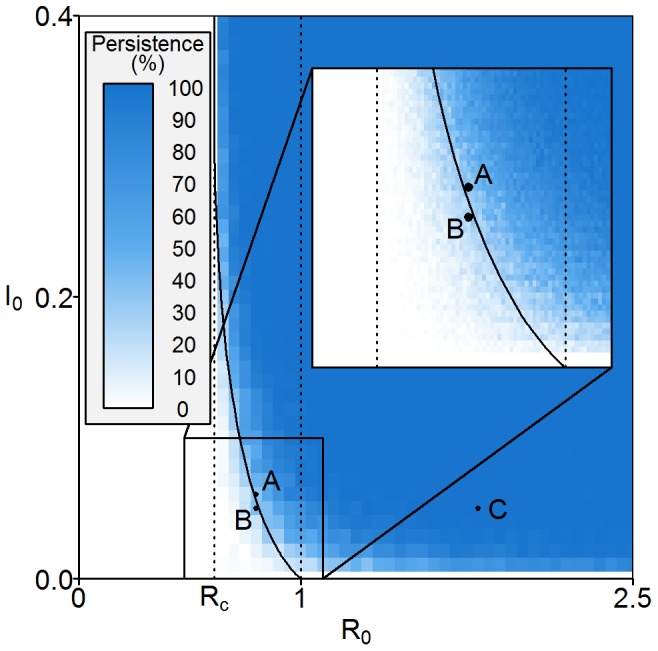
Persistence plot for the stochastic version of the explicit model. For given 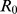 and 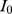 values, the plot shows the proportion of stochastic simulations that resulted in pathogen invasion and persistence (500 simulations per point). It can be seen that persistence is still determined by 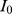 for a range of 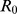 values (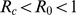) and furthermore that simulations with initial conditions below the deterministic threshold are still able to persist (see inset). Initial conditions for selected numerical simulations are shown. A:(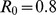, 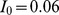) is just above the deterministic invasion and persistence threshold for the explicit model. B:(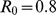, 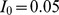) is just below the same threshold. C:(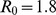, 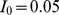) is significantly above the invasion and persistence thresholds for both models. The default parameter values are given in Table 2.

**Figure 8:**
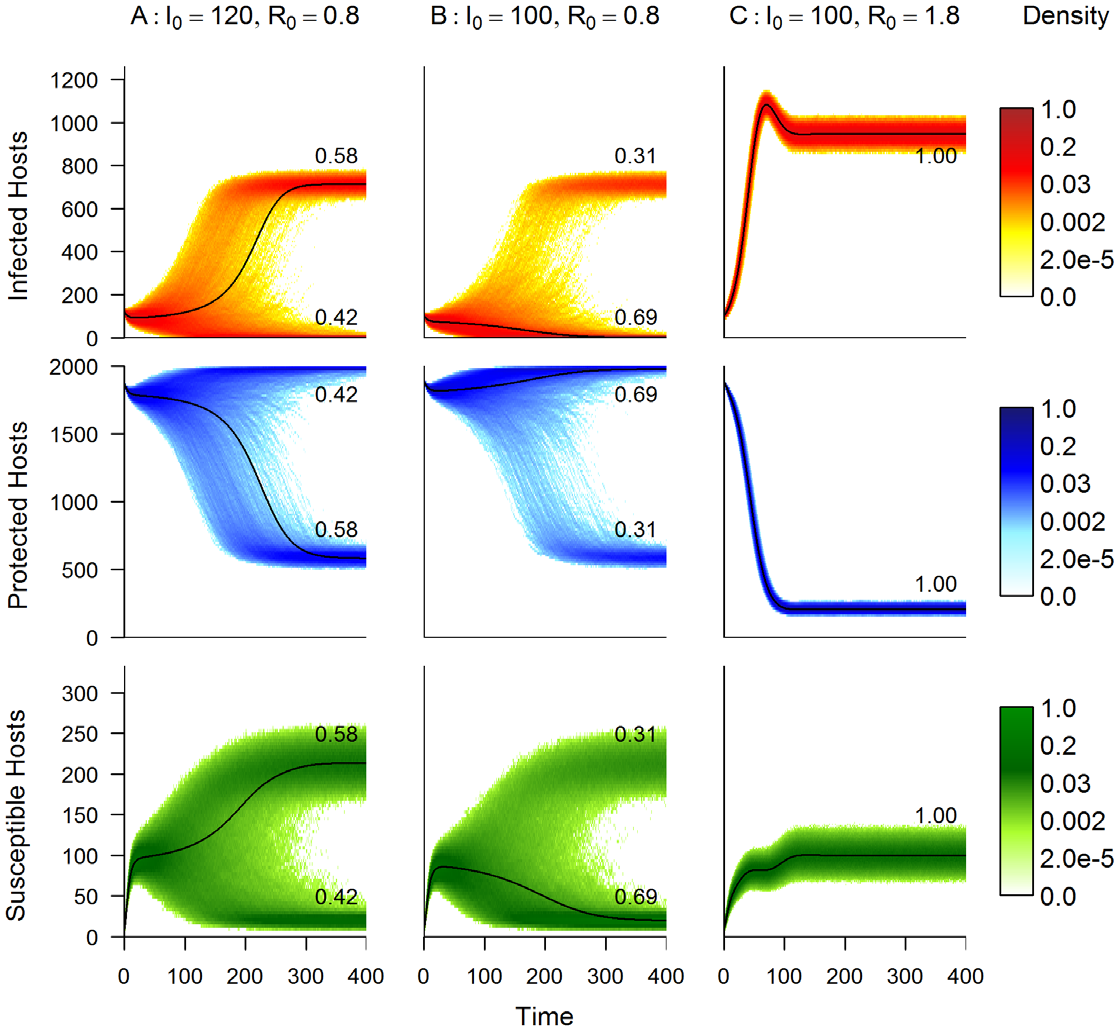
Probability density plots from the stochastic model. Here we show 5000 stochastic realisations of the explicit model that correspond to the initial condition points in the 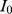-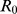 parameter space shown in Figure 7. Here the host carrying capacity, 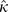 is 2000. The left hand figures show the probability density plots for all classes with initial conditions given by Point A (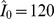, 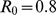) i.e. just above the deterministic invasion threshold, the middle column shows the probability density plots for Point B (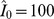, 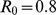), just below the invasion threshold, and the right hand column shows the probability density plots for Point C (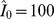, 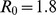), which is significantly above the invasion threshold. The deterministic trajectory for the corresponding class and initial condition is overlaid on each plot. The numbers within each plot indicate the proportion of realisations that reach those final distinct host levels. Note that the density scale is non-linear. The default parameter values are given in Table 2.

Using a nominal carrying capacity of 2000 (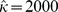), 5000 simulations are performed for every initial condition in the 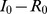 parameter space (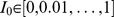, 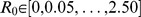, starting from a disease-free equilibrium level of uninfected hosts only) and the proportion of simulations that result in pathogen persistence is recorded and shown in Figure 7. The bistability zone still exists when allowance is made for demographic stochasticity. Figure 8 shows probability density plots obtained from the 5000 simulations for the initial conditions corresponding to points B (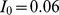, 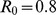), C (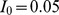, 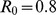) and D (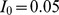, 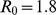). It can be seen that for a stochastic framework, starting at initial conditions near to the deterministic threshold, individual trajectories may tend towards either equilibrium (endemic or disease free) regardless of whether the initial condition is above or below the deterministic persistence threshold and so we see distinctly bimodal probability density functions as a result. This is due to demographic stochastic effects allowing individual trajectories to enter different basins of attraction and so tend towards either equilibrium. Crucially, this means that by creating a stochastic version of the explicit model we have shown that not only is the bistability zone still present but the range of initial infection levels that can lead to persistence is extended. The robustness of the effects was tested for a range of population sizes (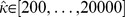) and shown to be sustained.

## Discussion

In this paper we develop a simple model to investigate the effects of fungicide dynamics on pathogen invasion and persistence. Specifically we incorporate protectant fungicide dynamics into a conventional model in a way that separates out the effects of partial coverage, incomplete effectiveness and decay in activity of a protectant fungicide to create an alternative model that takes explicit account of the fungicide dynamics. Previous work has utilised simple models of fungicide application to investigate a number of issues e.g. fungicide resistance [9], [13], [19], [30] and optimal control strategies [8]–[10], but the models used have not incorporated explicit fungicide dynamics (as the convention is to take implicit account of fungicide dynamics by mapping the effect onto the transmission rate).

The allowance for explicit fungicide dynamics markedly changes the inferences on both the invasion and persistence of the pathogen. Both frameworks appear superficially to have the same epidemiological properties and thresholds (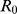 is identical for both) but the explicit model exhibits a bistability zone for values of 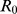 less than 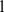, a range that the implicit model considers completely safe from invasion. Previous work on human and animal vaccination models [29], [31], re-infection models [32] and models of sexually transmitted diseases (with high and low transmissibility groups) [33] have exhibited similar bistability properties, but this is the first time that this property has been demonstrated for chemical control in agricultural systems. In addition previous epidemiological research has only considered the existence of a bistability zone for pathogens invading a system already at equilibrium. The extension of this result to systems where a pathogen invades a growing host state is a novel result with some striking repercussions. Not only does the inclusion of fungicide dynamics affect the criteria for invasion and persistence, (increasing the risk of a pathogen successfully invading and persisting) when compared with models that only implicitly include fungicide dynamics, but if the initial infection occurs at a time before the host population has reached its equilibrium state then this risk is exacerbated. Now it is even more likely that a pathogen will be able to invade and persist, and also there is an opportunity for a pathogen to simply invade in the short term, causing an early epidemic before eventually dying out.

The existence of both a bistability zone and an invasion zone below the traditionally accepted invasion threshold has several important implications both in the use of models to guide disease control, and in the selection of fungicide traits to promote effective disease control. Firstly, for a non-negligible, initial level of infection, a pathogen may be able to invade a system with a fungicidal control regime that an implicit model would predict to be adequate. For example, protectant application rates, fungicide efficacies and fungicide activity decay rates may be chosen to reduce the 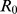 value of the system below 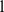 yet unknowingly still remain above the critical thresholds 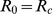, or 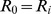. The system lies either in the bistability zone and is at risk from invasion by a high enough level of initial infection or it lies in the invasion zone and is at risk from an invasion during the host growth phase. The second implication is that for values of 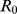 just above 1, the two models predict very different endemic levels of infection; for the implicit model, having an 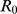 value just above 1 would always result in an invading pathogen achieving a negligible endemic equilibrium (see Figure 2) whereas for the explicit model an equivalent 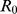 value will always lead to a much higher endemic equilibrium (see Figure 2). Consequently it would be possible for a system to experience sudden large invasions from small initial infections as a result of a small change in value of 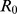 (from just below 1 to just above 1). Finally, in order to control an existing outbreak, it is necessary to reduce 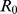 not just to below 1 but to below the critical threshold value 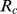. The predicted effort required to eliminate an established pathogen from a system therefore depends on the choice of model. A model without explicit fungicide dynamics will underestimate the effort required and consequently allow the pathogen to persist.

Of particular concern for disease management strategies is the drive in the agrochemical industry to produce longer lasting, more effective fungicides, which necessitate lower application rates. These aspirational fungicide properties are exactly those that correspond to the parameter region for the explicit model in which bistability zones occur (Figure 3).

A continuous-time, Markov process version of the explicit model is constructed and used to demonstrate that the bistability result is not just a property of the deterministic models. The simulations show that the probability distributions for the stochastic model exhibit bimodal behaviour in the region of bistability, with one mode at the endemic equilibrium and one at the disease free equilibrium. It is clear that the effects of fungicide dynamics on invasion and persistence criteria are still maintained when allowance is made for stochastic variability in the various transmission processes.

Taking all of this into account it is reasonable to conclude that failure to account for fungicide dynamics naturally leads to erroneous predictions of control effectiveness.
